# Sexualized Deepfakes in UK Schools: Understanding and Preventing AI-Generated Image-Based Sexual Abuse Through Better AI Literacies

**DOI:** 10.3390/bs16040554

**Published:** 2026-04-08

**Authors:** Jessica Ringrose, Tanya Horeck, Edith Rodda

**Affiliations:** 1 IOE, UCL’s Faculty of Education and Society, University College London, London WC1H 0AL, UK; 2 Cambridge School of Creative Industries, Anglia Ruskin University, Cambridge CB1 1PT, UK

**Keywords:** sexualized deepfakes, AI-generated image-based sexual abuse, tech-facilitated gender-based violence, AI literacy, teenagers, students, teachers, schools, adolescents

## Abstract

Responding to the lack of academic research on how young people are impacted by deepfake sexual abuse or how schools should address these issues, this paper explores levels of awareness of AI technology and sexualized deepfakes in UK schools and how schools are responding to these newly emergent harms. Drawing on interviews with students and teachers from eight schools across the UK, we found that teachers and students express uncertainty about how AI deepfake technology works. Some teachers underestimated how easy the technology is to use, and they lacked uniform comprehension that sexualized deepfakes should be treated the same way as non-consensual nudes, leading to inconsistency and variations in school responses. Students similarly lacked basic literacy about AI, equating AI with LLMs like ChatGPT, and even though sexualized deepfakes were occurring in their school contexts, students reported having received no explicit education on the topic. Educators and students connected sexualized deepfakes to a rise in misogyny via social media influencers, with some of the students and teachers calling for more education on AI, sexual violence, and consent at earlier ages. We advance the concept of AI-generated image-based sexual abuse, arguing that these harms should be understood as elements of technology-facilitated gender-based violence (TFGBV). We argue this framing is necessary to support systematic understandings of this issue and develop appropriate school responses. Our discussion offers recommendations for improving AI literacy, including preventative AI education that engages critically with AI harms and supports victims.

## 1. Introduction

Deepfakes are digitally altered content, which is fake, but depicts the faces, bodies, and/or voices of real people taken from other contexts. Deepfakes are part of a larger ecosystem of online harms facing young people in today’s 24/7 digital screen culture. AI-facilitated abuse is a growing problem, with reports of deepfake imagery being used maliciously against teachers and other students. Non-governmental organisations (NGOs) and charity groups internationally have documented the rise in sexualised deepfake abuse amongst youth.

The American technology non-profit Thorn (2025) released a stakeholder report surveying 1200 youth aged thirteen to twenty, finding that ‘one in 10 minors said they know of cases where their friends and classmates have created synthetic non-consensual intimate images (or “deepfake nudes”) of other kids using generative AI tools. Similarly, a Save the Children (2025) study of young people in Spain found that one in five participants reported that images of them naked had been created with AI when they were under eighteen, yet very few identified these experiences as abusive or illegal. In England, an Internet Matters (2024) report described AI-generated sexual imagery as an emerging ‘epidemic’. Their survey found that 13% of teenagers had experienced nude deepfakes in British schools. The report also found that 98% of deepfakes are sexual in nature (and 99% depict girls or women), yet 61% of children and 45% of parents reported that they do not know or understand the term ‘deepfake’. Further evidence from the Internet Watch Foundation (2025) indicates that 19% of confirmed reports of nude or sexual imagery of children and young people submitted to the UK’s Report Remove helpline involved digitally altered or manipulated content, including the use of AI or nudification apps. Finally, a Guardian report by Weale (2025), drawing on a Teacher Tapp survey, found that around one in 10 UK teachers were aware of students at their school creating ‘deepfake, sexually explicit videos’ in the previous academic year.

Coming from a preventative angle, these stakeholder reports emphasise the importance of conceptualising deepfakes from a sociotechnical perspective and establishing clearly that AI harms evolve not just from the digital world but also from gendered power dynamics (Internet Matters, 2024; Thorn, 2025; Save the Children, 2025). Importantly, the NGO research makes several recommendations for mitigating the risks posed by deepfakes to children in schools, noting the Relationship, Sex and Education curriculum as a vehicle through which AI literacy, consent, and digital ethics can be taught to students (Internet Matters, 2024).

In this paper, we use the term AI-generated image-based sexual abuse because it encapsulates the artificial intelligence technologies used as well as the sexual harm. As McGlynn and Toparlak (2025) note, it is important to acknowledge that the term ‘deepfake’ derives from ‘perpetrators of image-based sexual abuse’ and is problematic for that reason. We will also refer, however, to ‘deepfakes’ and ‘deepfake sexual abuse’, as these are commonly used terms which teachers and students are currently most familiar with. Terminology is important, and one of the critical things that needs to happen in schools is greater clarity around identifying and naming the forms of sexual harm caused by the misuse of AI technologies. Tech-facilitated sexual violence (TFSV) is defined as ‘a range of behaviours where digital technologies are used to facilitate both virtual and face-to-face sexually based harms’ (Henry & Powell, 2018, p. 195).

Dunn (2021, p. 25) uses the broader language of technology-facilitated gender-based violence (TFGBV), which encompasses gendered harms as well as sexual ones, and makes the all-important argument that technology-facilitated abuse is ‘actual violence’ and needs to be treated as such. We argue it is essential for AI-generated abuse to be conceptualised through the wider lens of TFGBV and defined as a specific form of non-consensual intimate image-based sexual abuse (Dunn, 2024) with real-life impact. Non-consensual sexual deepfakes need to be understood as a new iteration of tech-facilitated gender-based violence, as a form of image-based sexual abuse, which deploy AI technologies to produce fake, non-consensual images and videos of a sexualised nature. As McGlynn and Toparlak (2025) note: ‘The content almost exclusively targets women’ and ‘there has also been an exponential rise in “nudifying” apps which transform ordinary images of women and girls into nudes’.

To date, there is little academic empirical research on how teachers and students understand sexualised deepfakes or how schools are responding. There are several recent studies on TFGBV in secondary schools, which refer to the emerging threats of sexualised deepfakes. For instance, Ringrose et al. (2022, 2025) in the UK, Canada, and Ireland, have explored tech-facilitated violence, including the sharing of sexual images non-consensually, showing the lack of reporting and lack of adequate school responses to TFGBV, including several episodes of deepfakes. Ringrose and Regehr (2025) reported that boys use cut-and-paste technology to create fake dick pics to harass students, and that they receive no punishment. They found in their study that students received an inadequate curriculum on ethics, consent, and their digital rights, and experienced significant barriers to reporting abuse and harm either online or at school. Almanssori et al. (2025, p. 14) in Canada found that tech-facilitated gender-based violence was not understood or taken seriously in schools. Mentioning an example from 21-year-old student Elizabeth, which happened while she was in high school several years earlier, the perpetrator created a fake nude of her, but only received a 1-week suspension, with the episode traumatising Elizabeth beyond school years (Almanssori et al., 2025, p. 12) This research makes clear how continuing in these school settings is challenging, since they felt like ‘prisons’ where ‘threat of harm is omnipresent, [and] this fear shaped how students navigated relationships with peers and educators’ (Almanssori et al., 2025, p. 14). Critically, however, none of this research was conducted before the rise of nudify apps.

Thus, although most AI-generated abuse originates beyond the control of schools, schools have become a primary location in which these harms are recognised, interpreted, and managed. This means schools need to develop an adequate understanding and response to support young people. This situates educators at the intersection of technological risk, child protection, and gender–power relations, despite having limited training and no authority over the technologies themselves.

## 2. Methods

Against this backdrop we conducted focus groups with teachers and students from 8 secondary schools in England to explore their views, understandings, and responses to AI-generated image-based sexual abuse.

### 2.1. Research Questions

We aimed to gauge teachers’ and students’ understandings of AI sexualised deepfakes via the following research questions:

- What are teachers’ and students’ understanding of AI-generated image-based sexual abuse?
- What have teachers and students learned about AI-generated image-based sexual abuse in their school settings?
- How are schools responding to AI-generated image-based sexual abuse?
- How can schools better mitigate against AI-generated image-based sexual abuse moving forward?

### 2.2. Recruitment

We partnered with a school leadership organisation to recruit educators for our teacher focus groups and with a sexual violence charity to recruit the students we spoke to. Members of the sexual violence charity’s student advisory group volunteered to participate and were deemed most suitable to take part in the investigation, given they had received training on gender issues from the charity and had previously engaged in sensitive conversations.

### 2.3. Data Collection Procedure

We gathered the views of UK Teachers from 6 different schools (Table 1) via two online focus groups and an individual online interview (6 teachers in total). We gathered the views of students from two schools via one student focus group, composed of 4 girls from two independent schools (Table 2). In total, there were 10 participants from 8 different secondary schools.

Interviews were conducted in an online setting to enable participation. Each interview lasted approximately 60 min. A semi-structured interview guide was used to facilitate all the discussions, moving from broad introductory questions to specific probes regarding knowledge of AI and deepfakes. Different questions were directed towards the teachers, which touched on issues of school policies, whereas the student interviews were aimed at understanding their use and understanding of Gen AI across platforms.

One of our research team members led the interviewer while another took notes and participated with some key prompts. In the case of the student interview, alongside the two researchers, one of the charity’s school facilitators participated in the interview and did a debrief with the students following the interview in compliance with safeguarding around sensitive issues. All sessions were [audio/video] recorded with the explicit written consent of the participants.

While conventional guidance suggests that focus groups should ideally include between six and eight participants, there is no universal consensus on optimal group size, particularly in qualitative research involving sensitive or complex topics. Early focus group literature emphasises flexibility in design, noting that smaller groups may be more appropriate when discussing issues that require depth, trust, and careful facilitation (Morgan & Krueger, 1993). More recent methodological work similarly highlights that smaller groups—including those with as few as three participants—can be especially effective for complex or sensitive subject matter, as they enable more detailed discussion and reduce participant discomfort (Guest et al., 2017). In this study, given the highly sensitive nature of sexualised deepfakes and image-based sexual abuse, smaller group sizes were ultimately appropriate and, in some cases, preferable. This aligns with feminist methodological principles, which prioritise creating safe, non-hierarchical spaces that minimise power imbalances and support participants in sharing experiences of gender-based harm. Smaller groups enabled more in-depth discussion, reduced the risk of participants feeling exposed, and facilitated more ethically grounded engagement with a vulnerable population.

### 2.4. Ethics and Consent

We attained ethical clearance for the project, and followed strict ethical protocols, including safeguarding, informed consent, anonymity, and confidentiality. Prior to the interviews, all participants signed consent forms and returned them to us electronically afterwards. All data has been anonymised and pseudonyms are used throughout.

### 2.5. Data Analysis

The recordings were transcribed verbatim and anonymised to protect participant identity. Our analysis followed the thematic analysis framework developed by Braun and Clarke (2006), which involves a systematic process of coding and theme development.

First, transcripts were read repeatedly to enable familiarisation with the data. Second, initial codes were generated across the dataset, identifying recurring patterns, concepts, and meanings relevant to participants’ understandings and experiences of AI-generated image-based sexual abuse. These codes were then reviewed and organised into broader themes, which captured shared patterns across the interviews.

Themes were iteratively refined through ongoing comparison with the original data to ensure coherence, consistency, and analytical validity. In this way, coding functioned as a central step in structuring the analysis, enabling relationships between patterns in the data to be identified and interpreted. The themes presented in this paper reflect this process of coding, categorisation, and refinement.

In this paper, we explore three key themes that emerged from our analysis: 1. Students’ and teachers’ limited understandings of AI-generated image-based abuse; 2. Teachers and students situating deepfakes within a context of rising misogyny; 3. Inconsistent school responses and the need for better AI education.

## 3. Results

### 3.1. Students’ and Teachers’ Limited Understandings of AI-Generated Image-Based Abuse

In our research with girls from two UK independent schools, we found that as soon as we introduced Gen AI into the conversation, the students started discussing LLM’s like Chat GPT, which was their primary reference point for what constituted Gen AI:

Sam: Chat GPT … Um, I think it’s more of like a tool used in school. For me, I just use it to, like, help with sounds, like, weird but make essay plans and things like that, yeah?

In this excerpt, the students position AI firstly as Chat GPT (an LLM chatbot), which they use for homework and as a learning tool for tasks like designing ‘essay plans’. This framing suggests that these students primarily understand AI through school-sanctioned uses associated with learning, rather than as a technology capable of producing harm. While AI literacy appears to be developing in educational contexts, it is still narrowly defined, with limited awareness of the potential for misuse of generative tools.

After some prompting, Sam went on to note the rise of ‘fake videos’ on TikTok:

Sam: I get, like, a lot on my For You page and stuff with, like, just fake videos. Like, you can kind of tell they’re fake, but then, like, there’s ones you probably wouldn’t be able to tell that. You would see it anyway and absorb it as, like, thinking it’s real and it’s not and then there was, like, one incident with a boy in our year where, like, they edited his face onto, like, a naked woman. But I wouldn’t say that it’s like, like, I don’t prominently use it or anything like that. Like, I wouldn’t say it’s negatively affected me in any way in particular, but I know there’s, like, increased instances of use for it.

Although the students demonstrate an awareness of ‘fake videos’ circulating on social media, they did not initially connect this to deepfake abuse. The interviewer goes on to ask another student, Kate, how AI-generated content is coming up on specific social media like TikTok:

Kate: I think most of it is, like, I don’t call it brain rot, because, like, I don’t really, you know, engage with it, so it goes away quite quickly. Every now and again, like, one of those videos would come out … but then sometimes I get people saying, like, Oh, which one do you think is real? And it’s like, two handbags. And then I’m like, trying to guess, and I often get it wrong. So I think I interact with those ones a bit more because I find them quite interesting.

Kate is not aware of the programmes and platforms through which the deepfake videos they have seen on social media and around school are created, nor the algorithm that boosts this content as part of the TikTok recommender algorithm. She references ‘brain rot’, a term popularised in youth digital cultures to describe the consumption of repetitive, low-value online content (Oxford University Press, 2024). While colloquial, recent sociological work conceptualises ‘brain rot’ as a form of participatory practice within platform environments, tied to attention economies and the circulation of trivial or affective content (Owens, 2025). In this context, such content may contribute to the normalisation of synthetic media and desensitisation to its potential harms.

Kate’s account also highlights a disconnect between everyday encounters of synthetic media and the ability to recognise these practices as forms of IBSA. Engagement with ‘which one is real’ content suggests that synthetic media is often encountered as entertainment, rather than as something embedded in power, consent, or harm.

When asked directly about non-consensual sexual deepfakes, students demonstrated uncertainty about both terminology and technological processes. Amy recalled a personal development lesson discussing a reality television figure, but her account conflated deepfakes, leaked images, and revenge porn:

Amy: Yeah, they could, like, get AI to, like, take their clothes off, or, like, put them in another position, like a sexual position, or, I don’t know, I don’t know exactly what it can do.

This uncertainty suggests that even where schools have begun to address online harms, students lack the conceptual vocabulary to distinguish between different forms of image-based abuse. Students describe some patchy conversations they have had about editing faces and bodies (which seems like nudify apps/programmes), but they are confused if these are deep fakes or ‘deep leaks’, presumably the ‘leaking’ of nudes. We can see that they are not aware of the legal and technical terms of image-based abuse or deepfake abuse. These are huge gaps in their understanding around how technology is widely used, and even when conversations are taking place in schools, the correct technical language, laws, and responses do not appear to be readily available. Indeed, even though they have had dedicated lessons on these topics, clear information about the technology and how it can be used remains opaque.

Sam: We learn about … like the society online, and … like, AI photos of like Brad Pitt in hospital and like Brad Pitt, and this woman sent loads of money.

Kate: Like, do they do that with politics as well? They, like, AI generate politicians saying things, and then once they’ve said it, even if you know it’s fake, it still sticks in your brain. I think I’ve heard something like that.

Interviewer: Yeah, they can do deep fakes of people’s voices and also, like faces, and they’re really increasingly hard like to tell what’s real and what’s not.

Kate: What Sam was saying … I think I could be wrong, but I think she had shared a nude and then someone had, like, altered it, like they changed, like certain parts. So she still had shared the nude, but it was, like, made more I think. Georgia Harrison, I think, had the same thing and is now a motivational speaker, yeah.

The first example given refers to a ‘romance fraud’ in which scammers sent fake AI-generated selfies of Hollywood actor Brad Pitt sick in hospital, and tricked a French woman into sharing 830,000 Euros (Gozzi, 2025). The second refers to a criminal case of image-based sexual abuse, which is pejoratively known as ‘revenge porn’, but it is not an example of deepfakes or sexual digital forgeries. Students’ examples indicate that much of their understanding of AI-related harm is shaped by fragmented media narratives, rather than formal education.

This uncertainty reflects a broader lack of distinction between different forms of digitally mediated IBSA. While generative AI enables the creation of entirely synthetic images, other practices—such as editing, enhancement (e.g., using Photoshop), or redistribution of existing images—are often conflated with deepfakes. This lack of technical clarity is significant, as it obscures the specific affordances of AI-generated imagery, particularly its scalability, realism, and accessibility, and may limit young people’s ability to recognise the distinct risks and harms associated with these AI deepfakes. It also helps explain why students struggle to categorise their experiences, often drawing on fragmented media narratives rather than formalised conceptual frameworks. This aligns with existing research suggesting that young people’s understanding of AI remains limited and uneven, with less awareness of generative image technologies and their potential for misuse (Heeg & Avraamidou, 2025; Sanchez-Acedo et al., 2024).

When we spoke to teachers from six different schools in England about AI-generated image-based sexual abuse or ‘deepfakes’, we found they were scrambling to keep up with the rapid development of generative AI and what this means for learning at school, with the issue of deepfakes often being folded into the policies they were trying to establish. One senior leader explained that the school had introduced an AI policy, guidance for students, and curriculum audits, noting only briefly that ‘deep fakes have come into that’. This suggests that whole schools are beginning to institutionally respond to AI, and sexualized deepfakes are not yet consistently framed as a safeguarding or gender-based violence issue. Instead, they are subsumed within broader conversations about AI used in education.

Alan, a principal of a college that provides education for eleven- to sixteen-year-olds, suggested that society was ‘overestimating’ the technological ability of young people:

Alan: [When] we get into the world of deepfakes and such like, I think we slightly overestimate actually the technological ability of young people. I mean, there are some examples of some extremely advanced and clever young people, but there is something of a stereotype that, you know, young people are all super-duper wonderfully, you know, able to do things with AI and generative AI and so on, and they themselves are not necessarily quite all in that that sort of level of advanced sort of, you know creativity with it.

However, Alan then went on to describe multiple instances within his school in which students’ faces had been transposed onto pornographic images and circulated amongst peers. Alan disputes whether or not these images should be called ‘deepfakes’. In addition to cases of peer-on-peer AI-generated images, he described cases of ‘soft pornographic images of teachers, [their] bodies and faces transposed onto you know, people in various poses’. It is problematic that Alan fails to position these experiences of female staff as abusive. This contradiction is revealing. At the time of this conversation, in the summer of 2025, nudify apps were already accessible to young people, but these remarks from Alan suggest that educators were not necessarily aware of how easy they are to use. Alan’s comments also place the object of concern on young people, instead of the technology itself and adults.

Patricia, an assistant headteacher, similarly noted a disconnect between perceived and actual levels of understanding among students, explaining that many felt confident they could ‘navigate it’ and that ‘it’s going to be fine’, despite limited evidence of deeper knowledge. This perceived confidence is significant. It suggests that students may overestimate their ability to manage AI-related risks, which could further reduce the likelihood of seeking support from schools or reporting harmful experiences. Such overconfidence, combined with limited conceptual understanding, may contribute to a form of hidden vulnerability in which risks are normalised or minimised. It is also possible to speculate that students might be reluctant to involve the school in helping them to navigate AI, and feel they can sort out any issues that arise on their own. Research has consistently found that threats of punishment via zero-tolerance approaches to bullying and cyberbullying have resulted in underreporting of abuse from young people (Brunecz, 2015; Ringrose et al., 2021).

These findings align with emerging research on AI-generated image-based sexual abuse, which suggests that public and youth understandings of these harms remain limited and uneven. For example, recent work by Flynn et al. (2025) highlights significant variation in how individuals recognise and interpret AI-generated sexual imagery, with many participants struggling to identify such content as abusive or harmful. Similarly, research on image-based sexual abuse more broadly has found that while the circulation of non-consensual imagery is widespread, there is often a lack of shared understanding regarding its seriousness, legality, and impact (Umbach & Henry, 2025).

### 3.2. Teachers and Students Situating Deepfakes Within a Context of Rising Misogyny

Across the interviews, both teachers and students consistently situated sexualized deepfakes within a broader context of rising misogyny and online harm. Rather than understanding deepfakes as isolated technological incidents, participants framed them as part of wider patterns of gendered behaviour, particularly within peer cultures and digital environments.

Several teachers described an increase in harmful sexualised behaviour over recent years, often directed by boys towards girls and women. Alan, for example, positioned AI-generated imagery as part of a broader shift in online behaviour:

Alan: … over the last three or four years there has most certainly been a much higher level of online harmful behaviour, harmful sexual behaviour, online. And it’s mostly, but not exclusively, directed by boys towards girls and women and it’s some of it’s very kind of typical old school language so really the technology is just a conduit for expressing age-old, you know, tropes …

Alan expresses concern about the reversal of liberal, well-informed views amongst young men. He recognises that it is important to include boys in the conversation and not just demonise them:

Alan: As far as people’s understanding of what young boys and men are saying, doing and feeling, and that isn’t just a simple well we’ve got to tell them it’s all wrong. Because that doesn’t get you anywhere, just to do that, you’ve got to have conversations about why they are feeling this way, why they are, you know, showing these kinds of behaviours.

This framing positions deepfakes not as entirely novel phenomena, but as extensions of existing patterns of IBSA. Alan explains the need to have conversations with boys around issues like deepfakes, but does not articulate this in terms of addressing abuse and sexism in the same way some of the women teachers did. For example, Caroline, a teacher and PhD candidate, explicitly positions deepfakes in relation to misogyny:

Caroline: With the deep fakes, … it followed a period of increasing, I’m going to use the word misogyny because that’s what it was to staff and students.

Cindy, an assistant headteacher for Inclusion and Well-being for Key Stage 3 pupils at a school in Wales, and Erin, an associate executive principal at a group of special provision schools in the North of England, likewise discuss the rise in misogyny, racism, and far-right content in their school and community settings:

Cindy: Yeah, we’ve definitely seen a huge rise in misogyny and intolerance in general, actually. Also, racism—and we’re an inner city school—… we’re still seeing incidences of racism even between the non-whites. We’ll actually call each other racist names and things and it’s definitely a case of what we’re seeing in, in the community at large.

Erin: Yeah, I think you know, last summer was really concerning for us we’re not far from [area where anti-immigration protests erupted] which has hit the headlines quite a lot in the summer we know that children were accessing and shown a lot of content online round, you know, quite a lot of far right extreme views, and I think it’s again, it’s back to the algorithms, isn’t it?

Caroline, Cindy, and Erin are highlighting how platform environments are experienced as immersive and can be difficult to counter through education alone. While these accounts reflect participants’ perceptions rather than causal claims, they align with emerging research suggesting that recommender systems can amplify misogynistic and extremist content within youth digital spaces (Barker et al., 2024; Haslop et al., 2024).

Erin expressed concern about the accessibility of pornography online and how images are spread and shared amongst peer groups.

Erin: I’m quite aware that a lot of children access pornography from a young age … It’s very normalised …, I think, especially amongst boys. Readily available and you know they will share pornographic images, videos, etcetera … I think when they’re seeing it day in, day out it’s sometimes a challenge to have a counter narrative when there’s so much being thrown at them.

Erin’s proposition that pornography is ‘very normalised’ suggests that exposure to sexualised content is not exceptional, but embedded within everyday peer cultures and digital practices. Thus, rather than representing a radical break from existing practices, sexualised deepfakes can be understood as an extension of already normalised forms of image-sharing, peer-based circulation of explicit material.

The students, similarly, discussed how they are seeing a general rise in misogyny online and how this shapes their social worlds. Amy shared an unsettling story about how online misogynistic culture was affecting her own family:

Amy: I don’t want to talk too loudly, because my brother’s home, but he’s older than me, and he has come back from university, completely ultra-right, like, evil political, the most evil political arguments that you’ve ever heard, super like sexist arguments always highlights, like, it’s really horrible, but I know, like, what he’s listening to, I’ve heard it before. Like, all, like, the algorithm, yeah, say a video comes up with, like, Andrew Tate on my phone. I’m gonna watch most of it before I get, like, sick of it, because, purely just because I want to see, how can people actually believe this. Do you I mean, like, and I want to hear, like, what misinformation that they’ve been listening to stuff like that, even Andrew Tate, like Charlie Kirk, like those, really, just, like, really ultra right stuff, mostly about women and, like, all of this stuff’s coming up. … now he’s talking about abortion and all of this. It’s like two years ago, when he was at my school, not at University, he wasn’t like that. But you know, what’s he been watching like to make him like that? That’s what my mind goes to. But yeah, it’s definitely put a bit of a damper on the household.

Amy’s account illustrates how young people interpret shifts in attitudes and behaviour through the lens of digital media consumption. While her narrative does not establish direct causality, it highlights the perceived relationship between online content and changing gendered dynamics, and how these transformations are experienced in everyday life. Sam elaborated on this type of content, noting she felt that the boys in her school are not being indoctrinated into it in the way that Amy’s brother is, but are finding it more of a joke:

Sam: I feel like within our school, I think it’s more boys find it funny to say … I think a lot of them find it funny to make Andrew Tate jokes or say, like, quotes off of TikTok kind of thing. But I don’t actually think they’re really believing it.

Sam’s perspective complicates more linear narratives of influence by highlighting the role of humour, peer dynamics, and social performance in the circulation of misogynistic ideas, suggesting that engagement with such content may be more informed by the desire for group belonging than ideological commitment.

### 3.3. Inconsistent School Responses and Need for More AI Education

Both students and teachers positioned sexualized deepfakes as part of a wider ecosystem of online harm, yet responses within schools were often fragmented and inconsistent. Teachers described incidents involving AI-generated sexual imagery, but there was limited clarity around how such cases should be categorised or addressed institutionally. Caroline, for example, recounted a case in which students created and shared a deepfake in one of the schools she had taught in:

Caroline: Three boys in sixth form created a deepfake of a female student in a pornographic video, well a sexual video because yeah, pornographic suggests consent, and this was shared. And it was quite difficult at the time to determine what students had created versus shared it, so some students, it was kind of a “their word against our word,” and it was obviously very reactive.

Here, Caroline clearly articulates that such practices should be identified as abusive. She also puts the episode in the context of other forms of abuse taking place on social media involving the objectification and sexualisation of girls. However, while Caroline explicitly identified this as abusive behaviour, she also emphasised the difficulty schools face in establishing responsibility and responding consistently. The ambiguity surrounding creation, distribution, and intent complicates traditional disciplinary frameworks used by schools.

When we discussed what approaches could work to address these issues collectively, many of the teachers agreed that not enough was being done to educate on these topics. However, there were differing views on how to approach the issue of AI abuse and deepfakes. Alan described an incident in which a girl withdrew from school after a deepfake image was widely circulated:

Alan: We did manage eventually to turn it around and get her to … develop enough confidence again …, given these young people typically sort of [aged] 12/13/14, the most important thing for them is that they feel that they have support from their peers.

In this example, the school found peer support for the victim of AI-generated sexual harm to help make it possible for her to re-enter school. The focus is upon the individual girl rebuilding her confidence in the face of peer rejection, rather than framing this episode as one of AI-generated abuse. Alan lacks a clear understanding, as with some of the other teachers, that these images should be treated in the same way as ‘real’ non-consensual nudes. The response focuses on supporting the individual victim, rather than addressing the broader structural or cultural conditions underpinning the abuse.

Alan acknowledged how hard it is for victims to be in the same space as perpetrators at school, remarking on how fraught things can get when the ‘problem spills out into the physical world … of day-to-day life in school’. Describing how the situation was dealt with, Alan noted ‘consequences’ for perpetrators were implemented, ‘ranging from suspension through to internal consequences, perhaps you know losing social time and such like those sorts of typical things’. Alan also said that he has not permanently excluded anybody specifically for distributing deepfake images. He felt strongly that what was required was mediation with students, as well as parents:

Alan: What we don’t do is just plow in, you know, administer immediate discipline and then walk away because that’s not really addressing the problem. I mean, we’ll sit down with a young person, we do take a very firm and robust line on it and present to them is principally why they have done something, getting them to understand why what they’ve done is very harmful and very wrong and how it makes other people feel …

There are clear tensions in this response, given that there appears to be a lack of understanding that a sexualised deepfake should fall under the same guidelines as an actual nude image, something that has recently been recognised in law that applies to adults. The blurriness around what such images are and how they should be treated is evident.

Alan was also mindful of the complexities of involving parents and spoke of how dealing with parents regarding such issues can be tricky. Incidents often become inflamed through parental activity and discussion on WhatsApp groups—‘anti-social media’ as he quipped. Alan’s hesitation around parental involvement is understandable, but clear guidelines around how to treat these episodes are necessary.

Approaches to discipline vary widely across schools, revealing a lack of consensus about how to respond to AI-generated IBSA. Miranda explained her school has ‘a very clear policy of referring that straight to the police’ and that this was strongly communicated to the students. In contrast to the headteacher Alan, who felt that mediation would be more effective so that children learn empathy and how to adjust their digital behaviours, Miranda said that permanent exclusion would be her response to cases involving deepfakes in her school:

Interviewer: And what do schools do? What sort of sanctions do schools do if there’s a deep fake of a teacher?

Miranda: I mean, I think that would be sexual misconduct. I think it would be permanent exclusion for us.

Alan and Miranda’s contrasting approaches highlight a central tension in school responses: whether sexualized deepfakes should be addressed primarily through discipline and criminalization, or through education and relational intervention. The absence of clear guidance leaves schools to navigate these decisions independently, resulting in uneven and sometimes contradictory practices.

Teachers also expressed ambivalence around the efficacy of punitive responses in relation to deepfakes, tech-facilitated harms, and general behaviour at school. Caroline, for example, was concerned about the school’s responses to what would be categorised in school policy domains as ‘harmful sexual behaviour’ (Setty et al., 2024):

Caroline: The punitive approaches I found incredibly difficult to navigate, especially around, you know, misogyny towards staff, and to give you a very brief example, I was in a school a few months ago. I was in a long-term cover and I had some male students talk about giving oral, giving someone a blowjob. They said it to me twice. So I reported it in the sense that you know, they didn’t realise I knew it, but I did and I wanted … a restorative chat to them to explain that you know it’s not appropriate to say that to my face as a teacher. [But] they got suspended for a week and I wasn’t comfortable with that as a staff member. And again, I wasn’t involved in the decision and had I been, I would have explained that that wasn’t somewhere I wanted it to go. And I think as an educator, being in those settings, it can be very complicated when then the government is also saying zero tolerance.

Caroline’s discomfort with punitive behaviours reflects a disconnect between institutional disciplinary frameworks and teachers’ own pedagogical approaches, with her experience suggesting that zero-tolerance policies—while intended to signal seriousness—can produce responses that are disproportionate and do not consider the context of the behaviour.

She further reflected upon how boys’ abuse of one another was often taken less seriously:

Caroline: And it just makes me reflect on the other instances I saw at the school I was in last year … male students took pictures of a male student of his penis while he was going to the toilet and they threatened him to send it, but they [the school authorities] didn’t do anything, they just talked to them … but then there would be other instances where there’s AI deep fakes … and when it was a girl involved, it’s abuse. There were expulsions. When it was boys, it was, oh, it’s a laugh. I think there’s a tension there as well.

Caroline expresses concern here about how boys need to also be protected from image-based abuse, and how their experiences can be trivialised, which is consistent with research on boys’ and men’s failure to report abuse because of fears around response (Thomas & Kopel, 2023).

Cindy, an assistant headteacher for Inclusion and Well-being for Key Stage 3 pupils at a school in Wales, agreed that punitive and police responses could be ‘scary’ for young people:

Cindy: If you call police in, they will come in and do assemblies and things. For us, it’s very much our opinion though that we’re actually better at it than in some ways than the police are, because we can speak and get to the children in the way that police sometimes can’t, it can just be a little bit scary and a little bit shouty … when you’re dealing with anxious kids, that’s not necessarily the message you want to give them.

Similarly, Cindy expressed concern that involving the police could be counterproductive, particularly for ‘anxious kids’, suggesting that schools may be better positioned to engage students in ways that are supportive, rather than fear-based.

There is a range of opinions around how to best respond to these issues, some teachers suggesting a more ‘relational’ approach to work with children, while others suggest zero-tolerance approaches like permanent exclusion.

There was an inconsistency in the responses to sexualised deepfakes in schools, and almost no discussion of a curriculum that could be used to prevent these issues. Indeed, Caroline, who was not a school leader and worked as an educator in a more junior position, noted a disconnect with school leadership about how to broach these issues, leading to constraint in what she felt she could teach students about AI and deepfakes:

Caroline: I felt at times very confined by what I could speak about in terms of AI and deepfakes and there was a definite tension with what the teachers wanted to explore and what leadership wanted to explore and that kind of if you mention it, they may, you know, they’ll ‘go and use it’ type approach.

Caroline articulates a familiar worry that to speak to teens about issues of digital spaces, sexuality, relationships, and consent—in this case, AI and deepfakes— is understood by the school leadership as a problem, as it will give young people ‘ideas’ about how to use it. This chimes in with an abstinence message around digital harms like sexting, and simply means that young people are not given the information and skills to navigate these technological phenomena.

Students themselves recognised this gap and called for more comprehensive and earlier education. Amy, reflecting on the influence of misogynistic content on her brother and the challenges of addressing it, argued:

Amy: I guess the best way would be education like if this generation is going to be so bad at it, then at least let like four year olds be better educated on this like from the start. Like at younger ages we need more especially on consent and online safety, I think also I think they need more monitoring on most social media apps.

Both Caroline and Amy clearly argue that there is a need for more education and starting much earlier on these issues.

## 4. Discussion

### 4.1. Findings

Our research sought to understand how teachers and students understand and respond to sexualized AI deepfakes. Responding to our first research question ‘What are teachers and students’ understanding of AI-generated abuse?’, looking across the teachers and students, we can see both groups lacked understanding of deepfake technology and how to categorise sexualized deepfakes. The teachers demonstrated an acute lack of understanding of how AI technology works, how accessible deepfake technology is, and how easy it is to use. The students likewise lacked immediate understanding of AI-generated abuse. Their associations with AI were with chatbots like ChatGPT, which they use to assist with schoolwork, or fake videos on social media platforms like TikTok. This both sets up a precedent to accept LLMs in ways that could be problematic; there is a schism between the educational and entertainment elements of AI. The students lacked any insight into the technology being used to generate ‘fake videos’, which led them to lack insight into and minimise the examples of sexualized deepfakes they discussed in their school settings. It was only after an in-depth discussion that they started to link up experiences of seeing images of someone’s head pasted on a pornographic image as a coercive practice facilitated by AI.

In relation to our second research question—‘what learning about AI-generated abuse has happened in their school settings?’—both groups indicated there was a lack of any cohesive education to address these complex and interlinked forms of tech-facilitated gender-based violence in their schools. The students had never had explicit discussions of the technical elements of AI, which give it the capacity to alter and nudify images, nor had they discussed the issues of ethics and consent involved in these practices. Both the students and the teachers, however, connected sexualized deepfakes—and a lack of consent and ethics around nude imagery in general—to a rise in misogynistic influencers, which have normalised the abuse of women. This aligns with wider analyses of digitally mediated misogyny and its impacts on young people (Haslop et al., 2024; Bates, 2024). A couple of teachers explicitly connected this AI generation to a rise in other forms of misogyny in the school context towards female staff and students, and labelled the behaviour as abusive (Roberts & Wescott, 2024). One student referenced her personal life, noting her brother’s increased viewing of misogynistic influencers and a dramatic change in his behaviour towards women and girls since going to university. She called for greater education on these interconnected topics at an earlier age for young people. Young people also raised concerns about a rise in misogyny via specific social media influencers such as Andrew Tate, who have been identified in recent research as part of broader ‘manosphere’ ecosystems that circulate and normalise gendered harm (Haslop et al., 2024). The students believe that more education—and much earlier—was necessary to address these trends. Our findings chime with research about the increasing impacts of misogyny in schools and its effects upon teachers (Roberts & Wescott, 2024) and young people (Haslop et al., 2024), and calls for a comprehensive school curriculum and policies to address these issues.

Considering our third question about school responses to sexualized deepfakes, we found variation and inconsistencies in school responses. Several teachers related episodes of pasting students’ heads onto ‘pornographic images’, both of female students and staff. One of the students related a deepfake of a boy’s head pasted onto a woman’s body to shame him. It was worrying that some teachers lacked clarity on whether a sexualized deepfake should be treated the same as an ‘actual nude’. Cases were individualised, with the focus in one example on finding a way for the girl who was the victim of a widely circulated sexualized deepfake to re-enter school life and be accepted by her peers, rather than recognising or tackling the wider issue of AI-generated image-based sexual abuse in any type of policy or curricular way. Like Almanssori et al. (2025, p. 12), who found that the institutional rules and policies in schools ‘isolated … individual experiences rather than seeing these harms [as] reflect[ing] structural and institutional patterns in which technology amplifies pre-existing inequities’ our research found that the lack of understanding of how technology works, including ease of use and trends in AI, reduces the school’s ability to understand the abuse or support victims, who were discussed in terms of ‘rebuilding confidence’ rather than as sexual abuse survivors. Overall, we found contradictions and inconsistencies in school responses, and a clear need for better policy, teacher training, and support for young people and teachers in this area. The teachers lacked concrete guidance from their school about how to undertake education to mitigate against the types of non-consensual behaviour being discussed. There was an acute need for better AI media literacies that are attuned to the issues of sexualized deepfakes, consent, and abuse, and how to cope with episodes in supportive, ‘relational’ ways.

Responding to our final research question—‘How we can better understand and mitigate against these AI-generated harms and abuses in school settings moving forward?’—we explore future directions, offering some recommendations drawn from our research. Throughout our findings, we saw that teachers and students were connecting the malicious use of deepfakes to misogyny. Their discussion makes clear the need for an integrated education around algorithms, ethics, and issues of consent connected to AI. AI is not simply ChatGPT as they assume at first. It is part of a wider landscape of LLMs and algorithms that are shaping the direction of audiences and gendered viewing patterns online that cement misogyny in masculine peer groups (Barker et al., 2024). These dynamics dramatically impact the female students’ relationship with boys and young men, both in their families and in their relationships and experiences at school.

To address this, we advocate for a systematic strategy of tackling TFGBV in school communities (see Almanssori et al., 2025). In the UK, the PSHE (Personal, Social, Health and Economic) Association announced a new set of deepfake lesson plans for schools ‘to educate about deepfakes and protect children and young people from AI-generated sexual imagery’ (PSHE Association, 2026), but lesson plans need to be adopted and integrated through a systematic approach on this topic. The overall lack of prioritisation and preparedness to address AI-generated IBSA in schools underscores the need for a contextualised, ‘whole school’ educational approach (Gilsenan & Sundaram, 2025).

Responding to this, we argue that education on AI-generated abuse should be situated within a wider frame of TFGBV. Such education should foreground the ways in which digital platforms and AI tools are socially and culturally situated in contexts of gendered power. Young people must be equipped to not only identify an artificially generated image, but to understand how and why these images are produced, circulated, and consumed, and the gendered and sexualized hierarchies and dynamics embedded in these processes. Non-consensual sexualized deepfakes are not simply neutral technological phenomena; they extend existing patterns of sexual abuse and harassment into digital spaces. Education must therefore emphasise ethical reasoning, consent, and recognition of harm, rather than treating deepfakes solely as technical problems. Media literacy education in schools can be reconfigured to integrate these insights. To be effective, education must move beyond individualistic skills-based approaches to foster critical understandings of digital platforms and power asymmetries (Henry et al., 2020).

Research on technology-facilitated coercive control (TFCC) illustrates that young people can learn to distinguish abusive from non-abusive digital behaviours based on context, relational power, and consent, in the use of technology (Atiénzar-Prieto et al., 2025). Educational initiatives could incorporate ethical (post)digital sexual citizenship, teaching students to recognise not only harm to themselves but also their responsibilities to others (Setty et al., 2024). For instance, bystander education can be implemented through scenario-based learning, discussion of real-world cases, and engagement with narratives of consent and power, enabling young people to develop both critical and empathetic skills (Ringrose et al., 2025). As Livingstone et al. (2020) argue, children are not merely passive recipients of protection but active rights-bearing citizens in digital environments. Educational strategies that empower students to exercise agency and participate in solutions, rather than simply enforcing top-down restrictions, are recommended by this approach, and a gender sensitive lens is crucial.

Girls and women often show greater awareness of gendered power dynamics (Ging et al., 2024), highlighting the necessity for educational programmes to adopt gender-aware approaches that support raising awareness around harmful and healthy masculinities in relation to underreporting and supporting boys in what has been called greater awareness of ‘gender justice’—a critical component of tackling tech-facilitated gender-based violence (Keddie et al., 2023). These issues need to be addressed through the convergence of digital literacy and relationship and sexuality education in the school curriculum, so that it is given time and space. For instance, going through issues of consent in the use of AI to produce images is a key step in mitigating against this new form of AI-facilitated abuse. Issues related to trust and support when AI harms occur are also crucial.

### 4.2. Limitations

This study should be understood as exploratory pilot research in an emerging and highly sensitive area. This study is based on a sample of six educators and four students across eight secondary schools, and therefore does not and cannot produce generalizable findings about the UK school system. Due to the ethical complexities of researching sexualized deepfakes with young people, we partnered with a youth charity to support recruitment and safeguarding of participants. The student participants were members of a youth advisory group and had received prior training and support that enabled their participation in a sensitive study. While this did facilitate ethically robust and informed participation, it also means that the sample is not representative of the broader school population.

Additionally, the absence of male participants is a further limitation, given that boys were frequently identified by both students and teachers as key actors in the production and circulation of sexualized deepfakes. While the accounts presented here provide important insight into how these behaviours are experienced, perceived, and responded to by girls and educators, this study cannot speak to the motivations or dynamics that underpin boys’ engagement with these practices. This is particularly relevant given the emerging research on the role of masculinized online cultures and influencer ecosystems in shaping boys’ attitudes towards gender and sexuality (e.g., Haslop et al., 2024). Future research should therefore prioritise engaging boys and young men directly in order to better understand the conditions that influence their involvement in AI-facilitated harms, and to inform effective, gender-sensitive educational interventions.

As a pilot study conducted at an early stage in the public recognition of sexualized deepfakes, this research provides a foundational account of how schools and young people begin to make sense of AI-enabled harms as they emerge within everyday digital cultures. While the technological landscape continues to evolve rapidly, the issues identified in this study—particularly gaps in AI literacy, limited understandings of consent in relation to synthetic media, and inconsistent institutional responses—still pervade, and reflect enduring structural challenges in how educational settings engage with new forms of digital harm (Gerlich, 2025). As such, this study contributes a necessary empirical and conceptual foundation for ongoing research, policy development, and educational practices in this area. Further research should work to expand on this research with larger, more diverse samples and longitudinal approaches to track how awareness, policy, and educational responses continue to develop.

### 4.3. Implications for Policy and Practice

These findings demonstrate a need for practice-ready approaches that can be implemented within school settings. Recent policy and education guidance, including McGlynn and Toparlak’s (2025) work on sexual digital forgeries and new PSHE Association resources on deepfakes, emphasise the importance of recognising AI-generated non-consensual sexual imagery as a form of IBSA and responding accordingly. First, schools should explicitly classify AI-generated IBSA as within safeguarding and behaviour policies, ensuring that sexualized deepfakes are treated with the same seriousness as non-consensual intimate images. As McGlynn and Toparlak (2025) highlight, such practices constitute violations of privacy, dignity, and sexual autonomy, and should not be minimised as ‘harmless’ or ‘fake’ content. This directly addresses the lack of conceptual clarity identified in our findings, in which both students and educators struggled to categorise these incidents as abuse.

Secondly, AI literacy must be embedded within existing Relationships and Sex Education (RSE) and PSHE curricula. Newly developed PSHE guidance demonstrates that schools can meaningfully integrate teaching on AI-generated sexual imagery, including understanding the law, recognising harm, and knowing how to seek support (PSHE Association, 2026). In line with McGlynn and Toparlak’s (2025) call for clearer public understanding of sexual digital forgeries, this education should include explicit teaching on how generative AI tools function, how synthetic sexual imagery is produced, and the ethical and legal implications of creating and sharing this content. As our findings elucidate, the current educational landscape is fragmented, leaving students without the vocabulary to recognise or respond to harm.

Thirdly, schools should prioritise relational and restorative approaches when responding to incidents of deepfake abuse, particularly in cases involving minors, while maintaining clear recognition of harm. Our findings highlight tensions between punitive and relational responses, suggesting that zero-tolerance approaches alone may be insufficient to address the complexities of AI-facilitated abuse. Simultaneously, emerging guidance stresses the importance of preparing schools to respond proactively to incidents of deepfake abuse, for example, by having clear reporting pathways and support structures (PSHE Association, 2026).

Fourthly, schools require clearer guidance and targeted training for staff, including safeguarding leads and senior leadership, to ensure consistent and informed approaches to AI-generated harms.

Finally, whole-school approaches are needed to address deepfake abuse, which incorporate staff training, student education, and parent engagement, in addition to recognising that these harms emerge within broader sociotechnical environments shaped by platform design.

## 5. Conclusions

In this paper, we found a lack of understanding of AI-generated non-consensual sexual imagery in schools, both from teachers and teens. When it came to teachers, the lack of knowledge and awareness of how synthetic non-consensual intimate imagery constitutes a form of sexual abuse (like ‘real’ non-consensual nudes) led to inconsistencies in how to respond and support their students. With regard to teens, their confusion over the difference between AI-generated sexual abuse and image-based sexual abuse indicated a lack of education regarding definitions, language, and laws around AI-generated harms. Addressing these gaps, we argued there is an urgent need for a more joined-up discussion around issues of gender/sexual power imbalances, such as a rise in misogyny, in relation to AI-generated images and deepfakes. Moving forward, we recommend incorporating these understandings into more comprehensive AI media literacy aimed at upskilling young people and teachers on issues of ethics, privacy, and consent in their everyday uses of AI.

## Figures and Tables

**Table 1:** Educators who participated in the study.

Name	Role	School Type	Location
Alan	Principal	College	East Midlands
Caroline	English Teacher	State Comprehensive School	London
Patricia	Assistant Headteacher	Girls’ Grammar School	South-east England
Cindy	Assistant Headteacher	Comprehensive School	Wales
Erin	Associate Executive Principal	Specialist Provision School	North of England
Miranda	Headteacher	Girls’ Grammar School	South-west England

**Table 2:** Students who participated in the study.

Name	Year Level	School Type	Location
Sam	Year 12	Independent School	Essex
Jen	Year 12	Independent School	Essex
Amy	Year 12	Independent School	London
Kate	Year 12	Independent School	London

## Data Availability

The data presented in the study are available on request from the corresponding author due to ethical reasons, which might make the location of identities of the participants discoverable.
